# Measuring Wasting and Stunting Prevalence Among Children Under 5 Years of Age and Associated Risk Factors in Papua New Guinea: New Evidence From the Comprehensive Health and Epidemiological Surveillance System

**DOI:** 10.3389/fnut.2021.622660

**Published:** 2021-03-03

**Authors:** Bang Nguyen Pham, Vinson D. Silas, Anthony D. Okely, William Pomat

**Affiliations:** ^1^Population Health and Demography Unit, Papua New Guinea Institute of Medical Research, Goroka, Papua New Guinea; ^2^School of Health and Society and Early Start, Illawarra Health and Medical Research Institute, University of Wollongong, Wollongong, NSW, Australia

**Keywords:** wasting, stunting, children under 5 years of age, CHESS, Papua New Guinea

## Abstract

**Background:** Papua New Guinea (PNG) has undergone a significant health transition, with the prevalence of non-communicable diseases increasing. Many children under 5 years of age suffer from the burden of malnutrition. While wasting and stunting still remain high, children who are overweight and obese are reportedly increasing.

**Objective:** This study reports the prevalence of wasting, stunting, underweight, and overweight children under five in PNG and explores potential household and maternal socioeconomic factors associated with malnutrition.

**Method:** Data were drawn from the Comprehensive Health and Epidemiological Surveillance System (CHESS) in PNG. Height and weight were directly measured, and wasting, stunting, overweight, and underweight statistics were determined using the 2006 WHO Standard Growth Standards. Household and maternal factors were assessed with parent interviews conducted by trained data collectors. Multivariate logistic regression analyses were conducted to report associations between selected socioeconomic correlates and child malnutrition outcomes.

**Result:** The prevalence of wasting, stunting, underweight, and overweight children was 13.8, 46.5, 18.2, and 18%, respectively. Children from households with food shortage were more likely to be wasted than those from households without such an experience [OR: 1.43 (95% CI: 0.93–2.21)]. Children from the poor quintile were more likely to be stunted than those from the richest quintile [OR: 1.2 (95% CI: 0.79–1.82)]. Other factors associated with wasting included living in an urban vs. rural area [OR: 1.36 (0.77–2.4)], middle household wealth quintile vs. richest quintile [OR: 0.77 (0.38–1.55)], mothers in union with a man vs. mother unmarried or live in union [OR: 0.76 (0.4–1.42)], and male children vs. female [OR: 0.77 (0.53–1.11)]. Factors associated with stunting included residing in urban vs. rural areas [OR: 1.13 (0.8–1.6)], mother in union vs. single mother [OR: 0.86 (0.59–1.24)], and mothers with preparatory/elementary vs. mothers with vocational/college education [OR: 0.15 (0.02–1.01)].

**Conclusion:** An integrated approach is needed to comprehensively address the household socioeconomic factors at the household level, contributing to the improvement of child health and development in PNG.

## Introduction

### Nutrition at Global Level

Approximately 6.3 million children under 5 years of age (CU5) die every year, globally. About one-third of these deaths are associated with malnutrition conditions (1–3). While malnutrition among CU5 continues to be a significant population health issue in low and middle income countries (LMICs), new challenges have emerged alongside socioeconomic transition (4). This includes a shift from traditional food choices and low calorie diets to greater access to a variety of high calorie, processed foods.

The second Sustainable Development Goal (SDG) and its associated targets and indicators highlight the importance of monitoring the prevalence of stunting, wasting, and other forms of malnutrition among children under 5 years of age.

### Factors Associated With Child Nutrition in PNG

Malnutrition contributed to 36% of child deaths in hospitals in Papua New Guinea (PNG) in 2012 (5). Despite recent economic growth, malnutrition remains a child health issue in PNG (6, 7). Recent social changes have impacted food choices and eating habits among the population and CU5 in particular (8, 9). The interactions between disease episodes and child growth are well known in PNG. Infectious diseases are important risk factor for stunting among children (10). Associations between malaria and reduced growth in children have been reported previously, especially among children under 2 years of age. The higher incidence of respiratory tract infections may explain the impaired growth among children in the highlands (11). A similar negative effect on growth was found in association with intestinal helminthiasis in children in urban and rural areas. Diarrhea is well-documented in the literature as a contributor to stunting and is associated with the mal-absorption of nutrients, especially zinc (12).

Socioeconomic development is a further contextual factor, in which poverty is the major risk contributor to poor child health and development (13). Previous studies on nutrition-associated risk factors in PNG suggested that general economic development has not led to improved nutritional outcomes among children (14). Low intake of protein has been reported among children who lived in families working in traditional subsistent agriculture, and the impact of geographical location on child growth, especially for children in Eastern Highland Province (EHP) and Madang Province, has been highlighted (15).

Most socioeconomic factors associated with child growth were related to household socioeconomic status. This suggests that the availability and accessibility to a variety of foods at the household level could be key determinants of child growth in PNG. Parents' socioeconomic demographic characteristics such as mother's education, marital status, and occupation are also important predictors of the nutrition status of their children (16).

### Rationale of the Study

Data on malnutrition including wasting, stunting, underweight, and overweight CU5 remain limited in PNG (9). Very few studies have examined these indicators in relation to the country's current health and epidemiological transition where both under-nutrition and over-nutrition co-exist (17). Analysis of the 1982 National Nutrition Survey (NNS) provided an estimation of nutrition indicators based on the local definition of child growth standards, stunting, wasting, or underweight statistics were defined as >1 SD below the national mean of child participants (12). Hence the data generated were not available for comparison with international data. Most recent data reported by the 24-h surveillance pilot study conducted by PNGIMR among 4 year olds in the Goroka and Daulo districts in EHP in 2018 showed that about 75% of child participants were of normal weight, around 5% were underweight, and over 20% were overweight or obese (18). These data suggest that considerable shifts in child nutrition are occurring in PNG. Furthermore, little is known about risk factors associated with wasting and stunting in PNG.

In this study, we report on two key SDG indicators: the prevalence of stunting and wasting among CU5 (aged 0–59 months) and also the estimated prevalence of underweight and overweight children. We also examined associations between household socioeconomic status and mothers' socio-demographic characteristics and wasting and stunting. Our hypothesis was that household socio-economic status and maternal socioeconomic demographic characteristics were associated with malnutrition outcomes in CU5.

## Method

### Data Source

In this paper, we used data from the Comprehensive Health and Epidemiology Surveillance System (CHESS), operated by the Papua New Guinea Institute of Medical Research (PNGIMR). CHESS is a new generation population-based surveillance system with an electronic population database consisting of six components. (i) Household socioeconomic demographic data; (ii) children under 5 years of age; (iii) women of reproductive age, 15–49 years; (iv) men of working age, 15–64 years; (v) morbidity of patients seeking healthcare services at primary health facilities; and (vi) mortality of deceased persons who died in the communities. These data components are interlinked via unique household and individual identification codes, following the PNG national coding system issued by the National Statistics Office. The design of CHESS has been described elsewhere (19).

### Data Collection and Management

Data were collected from five major provinces of PNG: Port Moresby (POM, the National Capital), Central, Eastern Highlands (EHP), Madang, and East New Britain (ENB), covering children living in both urban and rural sectors in the period July–December 2018. Interviews were conducted in *Tok-Pisin*, the most common local language in PNG, by village-based data collectors, who were trained in interview skills and research methods before visiting households to interview parents/child caregivers, using the CU5 questionnaire, which was specifically designed to collect child health data.

The CU5 data component comprised eight modules, including a module on anthropometry, in which all eligible children's height and weight were measured, using a portable electronic scale and stadiometer in accordance with WHO anthropometric protocol (20). Length was measured in children younger than 24 months. All the anthropometric records were quality checked before being entered into the CHESS database, using a standard data entry template, developed on the MySQL/Process Maker platform.

### Dataset and Variables

Raw data sets were extracted from the CHESS database as Microsoft Excel spreadsheets and converted into SPSS (version 20.0) for analysis. CU5 data included variables on resident location (rural-urban sector), date of birth and date of interview, age (in months) and sex, and anthropometry (height and weight). Data on maternal socioeconomic demographic characteristics include marital status, highest education attainment, and maternal age. Household wealth index was calculated using selected household socioeconomic variables on housing characteristics, water and sanitation, and household assets, using the principal component analysis method (multi-dimension factor analysis). Household wealth index was then grouped into five quintiles from the 1st to the 5th quintile, in which each household was designated a corresponding category, representing the poorest, poor, middle, richer, and the richest. Details of this analysis have been discussed elsewhere (21).

### Definitions

In this study, we used the 2006 WHO Child Growth Standards as the reference population, which is expressed in standard deviations (SD) (or z-scores). Anthropometric data (height and weight), together with individual data on age (in month) and sex were converted into age- and sex-specific Z-scores, using the WHO Anthro software (20). According to this method, children who had missing data on either age, sex, weight, or height were not included in the conversion. Three new variables: weight-for-height, height-for-age, and weight-for-age z-scores were then created in the dataset.

#### Wasting

Wasting children were defined as those whose weight-for-height was >2 SDs below the median of the reference population. Moderately wasting was between −2 and −3 SDs while those who were >3 SDs below the median were classified as severely wasting.

#### Stunting

Children whose height-for-age was >2 SDs below the median of the reference population were classified as moderately or severely stunted. Those whose height-for-age was >3 SDs below the median were classified as severely stunted.

#### Underweight

Children whose weight-for-age was >2 SDs below the median of the reference population were considered as moderately underweight and those who were >3 SDs below the median were classified as severely underweight.

#### Overweight

Given the recent epidemiological shift in child contrition in PNG, we also calculated overweight prevalence among CU5. Overweight was defined as a weight-for-age between 2 and 3 SDs units above the median of the reference population (moderate) and severely overweight (obese) was defined as a weight-for-age >3 SDs above the median of the reference population.

These indicators were then stratified by urban-rural sector, province, child age group and sex, maternal education and marital status, household wealth quintile, and household food shortage experience (**Table 2**).

### Logistic Regression Analysis

To identify risk factors of child health and development, we conducted multinomial logistic regression analysis, in which stunting and wasting were treated as dependent variables for outcome measures of child health (the association of overweight and risk factors will be addressed in another paper). Socioeconomic demographic variables including child age groups (in months), sex of children, province, rural-urban sector, household wealth quintile and household experience of food shortage, mother's marital status, and mother's highest educational level attainment were used as dependent factors. Odds ratios (OR) were estimated for each category of these variables, with the last category being used as a referral group regarding the two outcome measures: wasting and stunting. Next, 95% confidence intervals (CIs) and *p*-values were calculated for every OR estimates to confirm the level of significance of association (**Table 3**). These analyses were conducted to address the question of whether household and maternal socioeconomic characteristics had an impact on the stunting and wasting status of children. Hence, the analyses provide a better understanding of child health and development in the broader context of socioeconomic determinants in PNG.

## Results

Data from 3,132 CU5 were included in the analysis, comprising 1,231 in Central Province, 758 in EHP, 289 in Madang, 654 in ENB, and 200 in POM.

### Household and Maternal Socioeconomic Demographic Characteristics of Child Participants

Table 1 shows household and maternal socioeconomic demographic characteristics of child participants. About 70% of children were from rural areas and 30% were from urban areas. The child distribution increased across age groups, from 6.0% in the youngest age group (0–5 months) to 27% in the oldest age group (48–59 months). More than 50% of the total children were male.

**Table 1:** Household socioeconomic and maternal demographic characteristics of children under 5 years of age by province, PNGIMR's CHESS, 2020.

			**Central**	**EHP**	**Madang**	**ENB**	**POM**	**All sites**
Sector	Urban	*N*	–	261	289	225	200	974
		%	–	34.4%	100.0%	34.4%	100.0%	31.1%
	Rural	*N*	1,231	497	–	429	–	2158
		%	100.0%	65.6%	–	65.6%	–	68.9%
Total		*N*	1,231	758	289	654	200	3,132
		%	100.0%	100.0%	100.0%	100.0%	100.0%	100.0%
Age group (month)	0–5	*N*	84	26	27	37	15	189
		%	6.8%	3.4%	9.3%	5.7%	7.5%	6.0%
	6–11	*N*	72	26	27	63	18	206
		%	5.8%	3.4%	9.3%	9.6%	9.0%	6.6%
	12–23	*N*	171	124	39	131	16	481
		%	13.9%	16.4%	13.5%	20.0%	8.0%	15.4%
	24–35	*N*	287	169	60	143	59	718
		%	23.3%	22.3%	20.8%	21.9%	29.5%	22.9%
	36–47	*N*	275	199	65	147	50	736
		%	22.3%	26.3%	22.5%	22.5%	25.0%	23.5%
	48–59	*N*	342	214	71	133	42	802
		%	27.8%	28.2%	24.6%	20.3%	21.0%	25.6%
Total		*N*	1,231	758	289	654	200	3,132
		%	100.0%	100.0%	100.0%	100.0%	100.0%	100.0%
Sex of child	Male	*N*	628	394	142	335	110	1609
		%	51.2%	52.4%	49.1%	51.8%	56.1%	51.7%
	Female	*N*	599	358	147	312	86	1502
		%	48.8%	47.6%	50.9%	48.2%	43.9%	48.3%
Total		*N*	1,227	752	289	647	196	3,111
		%	100.0%	100.0%	100.0%	100.0%	100.0%	100.0%
Age group of women	15–19	*N*	209	112	55	161	30	567
		%	25.3%	18.2%	19.2%	25.8%	22.7%	22.8%
	20–24	*N*	123	85	45	105	20	378
		%	14.9%	13.8%	15.7%	16.9%	15.2%	15.2%
	25–29	*N*	115	90	42	92	18	357
		%	13.9%	14.6%	14.7%	14.8%	13.6%	14.4%
	30–34	*N*	125	95	34	84	27	365
		%	15.1%	15.4%	11.9%	13.5%	20.5%	14.7%
	35–39	*N*	85	75	41	70	10	281
		%	10.3%	12.2%	14.3%	11.2%	7.6%	11.3%
	40–44	*N*	102	103	40	77	16	338
		%	12.3%	16.7%	14.0%	12.4%	12.1%	13.6%
	45–49	*N*	68	56	29	34	11	198
		%	8.2%	9.1%	10.1%	5.5%	8.3%	8.0%
Total		*N*	827	616	286	623	132	2,484
		%	100.0%	100.0%	100.0%	100.0%	100.0%	100.0%
Mother educational level	Preparatory/elementary	*N*	43	33	2	22	6	106
		%	6.5%	8.0%	0.7%	3.6%	5.9%	5.1%
	Primary	*N*	506	273	100	423	80	1382
		%	76.0%	66.4%	35.1%	69.2%	79.2%	66.6%
	Lower secondary	*N*	100	81	146	138	13	478
		%	15.0%	19.7%	51.2%	22.6%	12.9%	23.0%
	Upper secondary	*N*	11	16	25	16	1	69
		%	1.7%	3.9%	8.8%	2.6%	1.0%	3.3%
	Vocational/ college+	*N*	6	8	12	12	1	39
		%	0.9%	1.9%	4.2%	2.0%	1.0%	1.9%
Total		*N*	666	411	285	611	101	2,074
		%	100.0%	100.0%	100.0%	100.0%	100.0%	100.0%
Mother marital status	Married	*N*	611	327	131	285	104	1,458
		%	55.3%	52.8%	45.8%	45.6%	56.5%	51.7%
	With partner	*N*	109	106	45	69	20	349
		%	9.9%	17.1%	15.7%	11.0%	10.9%	12.4%
	Single	*N*	385	186	110	271	60	1012
		%	34.8%	30.0%	38.5%	43.4%	32.6%	35.9%
Total		*N*	1105	619	286	625	184	2,819
		%	100.0%	100.0%	100.0%	100.0%	100.0%	100.0%
Household wealth quintile	Poorest	*N*	294	255	–	20	54	623
		%	23.9%	33.6%	–	3.1%	27.0%	19.9%
	Poor	*N*	313	169	–	102	45	629
		%	25.4%	22.3%	–	15.6%	22.5%	20.1%
	Middle	*N*	245	114	3	151	43	556
		%	19.9%	15.0%	1.0%	23.1%	21.5%	17.8%
	Richer	*N*	259	147	49	202	36	693
		%	21.0%	19.4%	17.0%	30.9%	18.0%	22.1%
	Richest	*N*	120	73	237	179	22	631
		%	9.7%	9.6%	82.0%	27.4%	11.0%	20.1%
Total		*N*	1,231	758	289	654	200	3,132
		%	100.0%	100.0%	100.0%	100.0%	100.0%	100.0%
HH food shortage in the past 12 months	Yes	*N*	464	109	–	–	37	612
		%	37.8%	14.5%	–	–	19.0%	19.7%
	No	*N*	765	643	287	651	158	2,502
		%	62.2%	85.5%	100.0%	100.0%	81.0%	80.3%
Total		*N*	1,229	752	287	651	195	3,114
		%	100.0%	100.0%	100.0%	100.0%	100.0%	100.0%

More children of younger mothers than those of older mothers participated in the study, the highest proportion of mothers (about 25%) was in the youngest age group (15–19 years) and <10% were in the oldest age group (45–49 years). Most mothers (67%) reported obtaining a primary educational. Regarding current marital status, more than 50% of mothers reported that they were married, about 10% had partners, with more than one third (36%) reporting that they were single. Around 50% of the child participants in Central, EHP, and POM were from the poor and poorest households, compared to 20% in ENB, and zero in Madang. Nearly 20% of the households admitted food shortage in the past 12 months, with the highest proportion in Central (38%), followed by POM (19%) and EHP (15%), with negligible proportions in Madang and ENB.

### Prevalence, Trend, and Variation

Table 2 presents the estimated prevalence of wasting, stunting, overweight, and underweight child participants by selected socioeconomic demographic characteristics.

**Table 2:** Wasting, stunting, overweight and underweight prevalence among children under 5 years of age according to WHO Child Growth Standards by household socioeconomic and maternal demographic characteristics, PNGIMR's CHESS, 2020.

			**Weight for height**						**Height for age**						**Weight for age**					
			**Wasting**			**Overweight**			**Stunted**						**Underweight**					
			**Severe** **(< −3 SD)**	**Moderate** **(−3 to −2 SD)**	**Normal** **(−2 to +2 SD)**	**Moderate** **(2 to 3 SD)**	**Obese** **(>3 SD)**	**No. of** **CU5**	**Severe** **(< −3 SD)**	**Moderate** **(−3 to −2 SD)**	**Normal** **(−2 to +2 SD)**	**2 to 3** **SD**	**>3 SD**	**No. of** **CU5**	**Severe** **(< −3 SD)**	**Moderate** **(−3 to −2 SD)**	**Normal** **(−2 to +2 SD)**	**2 to 3** **SD**	**>3 SD**	**No. of** **CU5**
Sector	Urban	*N*	65	46	405	86	117	719	229	125	346	17	21	738	57	86	558	13	20	734
		%	9.0%	6.4%	56.3%	12.0%	16.3%	100.0%	31.0%	16.9%	46.9%	2.3%	2.8%	100.0%	7.8%	11.7%	76.0%	1.8%	2.7%	100.0%
	Rural	*N*	157	114	1,172	227	370	2,040	608	365	1,045	28	70	2,116	130	240	1,590	68	57	2,085
		%	7.7%	5.6%	57.5%	11.1%	18.1%	100.0%	28.7%	17.2%	49.4%	1.3%	3.3%	100.0%	6.2%	11.5%	76.3%	3.3%	2.7%	100.0%
Total		*N*	222	160	1,577	313	487	2,759	837	490	1,391	45	91	2,854	187	326	2,148	81	77	2,819
		%	8.0%	5.8%	57.2%	11.3%	17.7%	100.0%	29.3%	17.2%	48.7%	1.6%	3.2%	100.0%	6.6%	11.6%	76.2%	2.9%	2.7%	100.0%
Province^*^	Central	*N*	104	74	739	107	114	1,138	262	205	657	19	43	1,186	63	150	913	21	15	1,162
		%	9.1%	6.5%	64.9%	9.4%	10.0%	100.0%	22.1%	17.3%	55.4%	1.6%	3.6%	100.0%	5.4%	12.9%	78.6%	1.8%	1.3%	100.0%
	EHP	*N*	81	51	498	98	243	971	338	153	450	19	31	991	86	119	685	43	55	988
		%	8.3%	5.3%	51.3%	10.1%	25.0%	100.0%	34.1%	15.4%	45.4%	1.9%	3.1%	100.0%	8.7%	12.0%	69.3%	4.4%	5.6%	100.0%
	ENB	*N*	8	5	159	82	107	361	175	66	120	4	9	374	19	21	313	15	4	372
		%	2.2%	1.4%	44.0%	22.7%	29.6%	100.0%	46.8%	17.6%	32.1%	1.1%	2.4%	100.0%	5.1%	5.6%	84.1%	4.0%	1.1%	100.0%
	POM	*N*	29	30	181	26	23	289	62	66	164	3	8	303	19	36	237	2	3	297
		%	10.0%	10.4%	62.6%	9.0%	8.0%	100.0%	20.5%	21.8%	54.1%	1.0%	2.6%	100.0%	6.4%	12.1%	79.8%	0.7%	1.0%	100.0%
Total		*N*	222	160	1,577	313	487	2,759	837	490	1,391	45	91	2,854	187	326	2,148	81	77	2,819
		%	8.0%	5.8%	57.2%	11.3%	17.7%	100.0%	29.3%	17.2%	48.7%	1.6%	3.2%	100.0%	6.6%	11.6%	76.2%	2.9%	2.7%	100.0%
Sex	Male	*N*	84	61	683	140	207	1,175	366	200	583	19	42	1,210	74	134	921	32	40	1,201
		%	7.1%	5.2%	58.1%	11.9%	17.6%	100.0%	30.2%	16.5%	48.2%	1.6%	3.5%	100.0%	6.2%	11.2%	76.7%	2.7%	3.3%	100.0%
	Female	*N*	97	64	609	120	197	1,087	318	189	561	23	34	1,125	81	138	829	37	28	1,113
		%	8.9%	5.9%	56.0%	11.0%	18.1%	100.0%	28.3%	16.8%	49.9%	2.0%	3.0%	100.0%	7.3%	12.4%	74.5%	3.3%	2.5%	100.0%
Total		*N*	181	125	1,292	260	404	2,262	684	389	1,144	42	76	2,335	155	272	1,750	69	68	2,314
		%	8.0%	5.5%	57.1%	11.5%	17.9%	100.0%	29.3%	16.7%	49.0%	1.8%	3.3%	100.0%	6.7%	11.8%	75.6%	3.0%	2.9%	100.0%
Age group (month)	0–5	*N*	11	6	69	9	18	113	24	26	61	3	1	115	5	11	93	2	3	114
		%	9.7%	5.3%	61.1%	8.0%	15.9%	100.0%	20.9%	22.6%	53.0%	2.6%	0.9%	100.0%	4.4%	9.6%	81.6%	1.8%	2.6%	100.0%
	6–11	*N*	8	9	70	13	23	123	29	27	66	1	2	125	2	18	100	2	3	125
		%	6.5%	7.3%	56.9%	10.6%	18.7%	100.0%	23.2%	21.6%	52.8%	0.8%	1.6%	100.0%	1.6%	14.4%	80.0%	1.6%	2.4%	100.0%
	12–23	*N*	21	13	175	34	48	291	87	54	139	8	11	299	17	43	217	9	12	298
		%	7.2%	4.5%	60.1%	11.7%	16.5%	100.0%	29.1%	18.1%	46.5%	2.7%	3.7%	100.0%	5.7%	14.4%	72.8%	3.0%	4.0%	100.0%
	24–35	*N*	42	36	286	60	74	498	136	87	266	11	14	514	32	53	403	11	11	510
		%	8.4%	7.2%	57.4%	12.0%	14.9%	100.0%	26.5%	16.9%	51.8%	2.1%	2.7%	100.0%	6.3%	10.4%	79.0%	2.2%	2.2%	100.0%
	36–47	*N*	41	22	284	54	87	488	159	85	226	10	24	504	39	59	378	12	9	497
		%	8.4%	4.5%	58.2%	11.1%	17.8%	100.0%	31.5%	16.9%	44.8%	2.0%	4.8%	100.0%	7.8%	11.9%	76.1%	2.4%	1.8%	100.0%
	48–59	*N*	38	34	311	58	94	535	160	81	297	5	16	559	34	61	419	17	18	549
		%	7.1%	6.4%	58.1%	10.8%	17.6%	100.0%	28.6%	14.5%	53.1%	0.9%	2.9%	100.0%	6.2%	11.1%	76.3%	3.1%	3.3%	100.0%
Total		*N*	161	120	1,195	228	344	2,048	595	360	1,055	38	68	2,116	129	245	1,610	53	56	2,093
		%	7.9%	5.9%	58.3%	11.1%	16.8%	100.0%	28.1%	17.0%	49.9%	1.8%	3.2%	100.0%	6.2%	11.7%	76.9%	2.5%	2.7%	100.0%
HH wealth quintile	Poorest	*N*	43	34	311	46	74	508	121	87	296	5	14	523	36	66	383	14	17	516
		%	8.5%	6.7%	61.2%	9.1%	14.6%	100.0%	23.1%	16.6%	56.6%	1.0%	2.7%	100.0%	7.0%	12.8%	74.2%	2.7%	3.3%	100.0%
	Poor	*N*	49	33	268	54	73	477	144	82	244	9	16	495	31	71	364	9	12	487
		%	10.3%	6.9%	56.2%	11.3%	15.3%	100.0%	29.1%	16.6%	49.3%	1.8%	3.2%	100.0%	6.4%	14.6%	74.7%	1.8%	2.5%	100.0%
	Middle	*N*	24	23	229	48	74	398	124	71	197	10	13	415	26	39	317	13	13	408
		%	6.0%	5.8%	57.5%	12.1%	18.6%	100.0%	29.9%	17.1%	47.5%	2.4%	3.1%	100.0%	6.4%	9.6%	77.7%	3.2%	3.2%	100.0%
	Richer	*N*	31	19	261	57	74	442	141	78	208	9	19	455	25	47	365	9	8	454
		%	7.0%	4.3%	59.0%	12.9%	16.7%	100.0%	31.0%	17.1%	45.7%	2.0%	4.2%	100.0%	5.5%	10.4%	80.4%	2.0%	1.8%	100.0%
	Richest	*N*	14	11	126	23	49	223	65	42	110	5	6	228	11	22	181	8	6	228
		%	6.3%	4.9%	56.5%	10.3%	22.0%	100.0%	28.5%	18.4%	48.2%	2.2%	2.6%	100.0%	4.8%	9.6%	79.4%	3.5%	2.6%	100.0%
Total		*N*	161	120	1,195	228	344	2,048	595	360	1,055	38	68	2,116	129	245	1,610	53	56	2,093
		%	7.9%	5.9%	58.3%	11.1%	16.8%	100.0%	28.1%	17.0%	49.9%	1.8%	3.2%	100.0%	6.2%	11.7%	76.9%	2.5%	2.7%	100.0%
HH food shortage	Yes	*N*	52	33	314	41	59	499	116	86	290	6	20	518	32	74	383	10	17	516
		%	10.4%	6.6%	62.9%	8.2%	11.8%	100.0%	22.4%	16.6%	56.0%	1.2%	3.9%	100.0%	6.2%	14.3%	74.2%	1.9%	3.3%	100.0%
	No	*N*	109	86	874	187	283	1,539	476	272	759	32	48	1,587	97	169	1,219	43	39	1,567
		%	7.1%	5.6%	56.8%	12.2%	18.4%	100.0%	30.0%	17.1%	47.8%	2.0%	3.0%	100.0%	6.2%	10.8%	77.8%	2.7%	2.5%	100.0%
Total		*N*	161	119	1,188	228	342	2,038	592	358	1,049	38	68	2,105	129	243	1,602	53	56	2,083
		%	7.9%	5.8%	58.3%	11.2%	16.8%	100.0%	28.1%	17.0%	49.8%	1.8%	3.2%	100.0%	6.2%	11.7%	76.9%	2.5%	2.7%	100.0%

* *No Z-scores were generated for children in Madang by the WHO Anthrop software program because the anthropometric data were either missing or ineligible*.

Wasting prevalence was 13.8%, including 8% severe and 5.8% moderate. Wasting prevalence was slightly higher in the urban sector than the rural sector, 15.4 and 13.3%, respectively. Children in POM had the highest prevalence of wasting and children in ENB had the lowest. The difference between male and female children in moderate wasting was significant. There was no clear pattern of wasting across the child age groups. However, wasting was significantly higher among children from poor households than those from better-off households. For example, 15.2% were in the poorest and 17.2% in the poor compared to 11.3% in the richer and 11.2% in the richest household wealth quintile. Household food shortage was an important factor contributing to increased wasting. Seventeen percent of children who reported lack of food in the past 12 months were wasting, compared to 12.7% of children from households without food shortages.

**Figure 1:**
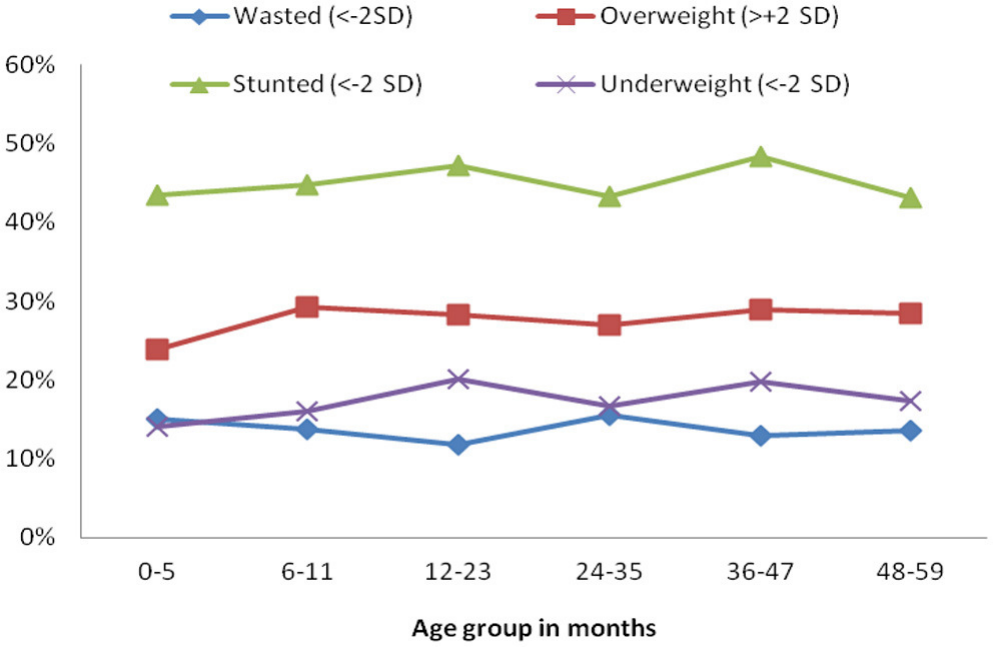
Percentage of children under 5 years of age, who are undernourished by age group in PNG, PNGIMR's CHESS 2020.

Stunting prevalence was 46.5%, with a non-significant difference between urban and rural children. Severe stunting varied widely across provinces, with the highest prevalence in ENB (46.8%), followed by EHP (34.1%), and the lowest in POM (20.5%). Two different patterns in stunting by age groups were observed; severe stunting was likely to increase with increasing age whereas moderate stunting tended to decrease as age increased (Figure 1).

**Figure 2:**
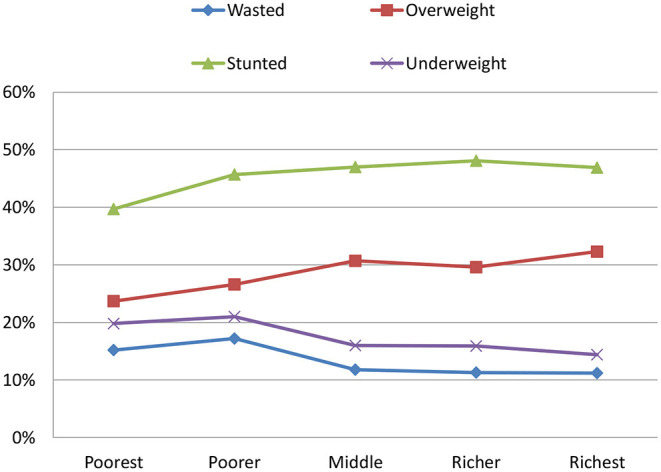
Percentage of children under 5 years of age, who are undernourished by household wealth quintile in PNG, PNGIMR's CHESS 2020.

Underweight prevalence among CU5 was estimated at 18.2%, with a non-significant difference between urban and rural children. A pattern of increased severely underweight children by age group was observed, with the lowest level (1.6%) in 6–11 months and the highest level (7.8%) in 36–47 months. By contrast, moderately underweight children fluctuated around 10–11% across age groups. The trend of decreased underweight children by household wealth quintiles was clear, with the highest prevalence of severe and moderate (7 and 12.8%) among children from the poorest households, and the lowest among children from the richest households (4.8 and 9.6%) (Figure 2). Comparing underweight statistics between children from households that experienced food shortage in the past 12 month and those without such an experience, the number of severely underweight children was similar between the two groups, but the number of moderately underweight children was significantly higher in the former compared with the latter, 14.3 vs. 10.8%, respectively.

Overweight prevalence was 29% with similar results between urban and rural sectors. The number of overweight children was noticeably highest in ENB (above 50%), followed by EHP (above 35%), and lowest in Central and POM (17–19%). The number of overweight children was similar between male and female children, 29.5 and 29.1%, respectively. There was no clear trend of moderately overweight children, but obesity increased by household wealth quintiles, from the lowest level of 14.6% in the poorest to 18.6% in middle, and 22% in the richest. The amount of overweight children was significantly higher among children whose families reported no food shortage in the last 12 months (30%) than those who reported a lack of food (20%).

### Associated Risk Factors for Wasting and Stunting

Associations between household maternal socioeconomic demographic factors and child wasting and stunting are shown in Table 3.

**Table 3:** Household socioeconomic and maternal demographic factors associated with wasting and stunting among children under 5 in PNG, PNGIMR's CHESS 2020.

		**Wasting status (< −2 SD)**						**Stunting status (< −2SD)**					
		***N***	**Percentage**	***P*-value**	**Odds ratio**	**Lower bound**	**Upper bound**	***N***	**Percentage**	***P*-value**	**Odds ratio**	**Lower bound**	**Upper bound**
Sector	Urban	274	23.0%	0.29	1.36	0.77	2.40	281	22.9%	0.49	1.13	0.80	1.60
	Rural	919	77.0%		Ref.			944	77.1%		Ref.		
Province	Central	537	45.0%	0.76	0.87	0.35	2.15	552	45.1%	0.26	1.43	0.77	2.67
	EHP	345	28.9%	0.90	1.05	0.48	2.30	349	28.5%	0.05	1.75	0.99	3.10
	ENB	237	19.9%	0.00	0.20	0.07	0.58	248	20.2%	0.00	3.29	1.80	6.01
	POM	74	6.2%		Ref.			76	6.2%		Ref.		
Household wealth quintile	Poorest	229	19.2%	0.67	1.16	0.59	2.28	233	19.0%	0.97	0.99	0.64	1.54
	Poor	274	23.0%	0.51	1.25	0.65	2.37	281	22.9%	0.39	1.20	0.79	1.82
	Middle	252	21.1%	0.46	0.77	0.38	1.55	260	21.2%	0.53	1.14	0.75	1.74
	Richer	286	24.0%	0.90	0.96	0.50	1.85	296	24.2%	0.70	1.08	0.72	1.63
	Richest	152	12.7%		Ref.			155	12.7%		Ref.		
Food shortage	Yes	259	21.7%	0.11	1.43	0.93	2.21	265	21.6%	0.51	0.90	0.67	1.23
	No	934	78.3%		Ref.			960	78.4%		Ref.		
Mother marital status	Married	536	44.9%	0.26	1.25	0.85	1.86	549	44.8%	0.89	1.02	0.79	1.31
	With partner	156	13.1%	0.39	0.76	0.40	1.42	162	13.2%	0.42	0.86	0.59	1.24
	Single	501	42.0%		Ref.			514	42.0%		Ref.		
Mother education attainment	Preparatory	23	1.9%	0.24	0.17	0.01	3.29	25	2.0%	0.05	0.15	0.02	1.01
	Elementary	52	4.4%	0.26	0.24	0.02	2.91	52	4.2%	0.24	0.35	0.06	2.01
	Primary	883	74.0%	0.67	0.62	0.07	5.53	901	73.6%	0.17	0.31	0.06	1.66
	Lower secondary	196	16.4%	0.78	0.73	0.08	6.67	203	16.6%	0.16	0.30	0.06	1.62
	Upper secondary	24	2.0%	0.95	0.93	0.08	10.43	27	2.2%	0.62	0.63	0.10	3.94
	Vocational/College+	15	1.3%		Ref			17	1.4%		Ref.		
Child age group in month	0–5	65	5.4%	0.66	0.81	0.32	2.06	65	5.3%	0.94	0.98	0.56	1.71
	06–11	77	6.5%	1.00	1.00	0.43	2.33	79	6.4%	0.97	1.01	0.60	1.69
	12–23	159	13.3%	0.40	1.29	0.71	2.34	165	13.5%	0.86	0.97	0.66	1.42
	24–35	284	23.8%	0.26	1.34	0.81	2.23	290	23.7%	0.89	1.02	0.74	1.43
	36–47	298	25.0%	0.81	0.94	0.54	1.61	307	25.1%	0.03	1.44	1.04	1.99
	48–59	310	26.0%		Ref.			319	26.0%		Ref.		
Sex of child	Male	620	52.0%	0.16	0.77	0.53	1.11	635	51.8%	0.63	0.95	0.75	1.19
	Female	573	48.0%		Ref.			590	48.2%		Ref.		
Total valid no. of children		1193	100.0%					1225	100.0%				
Subpopulation		808^*^						827^**^					

* *The dependent variable has only one value observed in 756 (93.6%) subpopulations*.

** *The dependent variable has only one value observed in 684 (82.7%) subpopulations*.

Children in urban areas were more likely to be wasting than those in rural areas [OR: 1.36 (95% CI 0.77–2.4)]. Children in ENB were less likely to be wasting than those from POM [OR: 0.20 (95% CI 0.07–0.58)]. Children from households that experienced food shortage in the past 12 months were more likely to be wasting than those from households without such an experience [OR: 1.43 (95% CI 0.93–2.21)]. Children from households of middle wealth quintile were less likely to be wasting than those from the richest wealth quintile [OR: 0.77 (95% CI 0.38–1.55)]. Sex of the child was an important factor associated with wasting among children, with male children less likely to be wasting than their female counterparts [OR: 0.77 (95% CI 0.53–1.11)].

Children from Central, EHP, and ENB were all more likely to be stunted than those from POM, with ORs of 1.43 (0.77–2.67), 1.75 (0.99–3.10), and 3.29 (1.80–6.01), respectively. Children from a poor household wealth quintile were more likely to be stunted than children from the richest quintile [OR: 1.2 (0.79–1.82)]. Children whose mothers lived with partners were less likely to be stunted than those whose mothers were currently not married or did not have partners [OR: 0.86 (0.59–1.24)].

## Discussion

Malnutrition encompasses wasting, stunting, being underweight, and even overweight in some cases, and can have both short and long-term impacts on child health and development (4). These impacts are well known to be associated with poor educational performance in childhood, lost productivity in adulthood, and increased risks of non-communicable diseases later in life (22).

As shown in Table 4, the 2005 PNG National Nutrition Survey (NNS) reported the prevalence of wasting among children aged 6–59 months at 4.5%, stunting at 43.9%, and being underweight at 18.1% (23). The 2010 HIES estimated wasting among CU5 at 15.8%, stunting at 46%, and being underweight at 25% (7). More recently, the National Health Information System (NHIS) reported that the prevalence of being underweight among CU5, who attended maternal and child health clinics, was 20% in 2018 (24). Our estimates support these figures, with the current numbers of wasting among CU5 at 16%, stunting at 46%, and being underweight at 18%. The data trend suggest that wasting is likely to have increased by more than 10% and there has been little improvement in stunting and being underweight among CU5 over the period 2005–2020, if the estimates of the 2005 NNS data are considered as reliable. It is noticeable that our study indicates a wasting prevalence (16%) higher than that reported by the 2005 NNS (4.5%), but it is similar to the level detected by the 2010 HIES. These figures suggest that wasting prevalence among CU5 had increased most likely in the period 2005–2010, but no further increase in the period 2010–2020. According to our knowledge and fieldwork experience, there is no clear socioeconomic or technical reason possibly explaining these trends. Furthermore, we have estimated that 28% of CU5 were overweight, for which the three other national data sources failed to report. This indicator is particularly important in the current health and epidemiological transition in PNG (25, 26).

**Table 4:** Comparison of health and development indicators among children under 5 years of age across different data sources.

	**2005 NNS** **(published 2011) (23)**	**2010 HIES** **(published 2015) (7)**	**2018 NHIS** (published 2019)^*^ (24)	**2019 CHESS** **(published 2020)**
Wasting	4.5%	15.8%	N/A	16%
Stunting	43.9%	46%	N/A	46%
Underweight	18.1%	25%	20%	18%
Overweight	N/A	N/A	N/A	28%

* *National Health Information System reported underweight among CU5 attended Maternal and Child Health Clinics*.

For the first time, we measure and report all four key nutritional indicators of PNG children against the 2006 WHO Child Growth Standards. Our data uncover an alarming level of wasting as well as overweight CU5 in PNG. We call for urgent action to improve the nutrition status among PNG children and to achieve the SDGs by 2030 as part of PNG's government commitments. Compared to other Pacific countries, our estimates of stunting among CU5 in 2020 (46%) are higher than that of the Solomon Islands in 1989 (34%), Indonesia in 2007 (40%), but lower than Timor-Leste in 2009 (58%) (27).

Our data show a complicated picture of nutrition among CU5 in PNG, in which multiple factors interact to cause child malnutrition. Examining possible associations with various household, maternal, and individual socioeconomic demographic factors, we found that wasting and stunting are distinct phenomena with different correlates. These two issues could share the same common socioeconomic determinants, but maternal socioeconomic demographic factors appear to be different in the development of wasting and stunting.

Wasting may exhibit significant seasonal shifts associated with recent changes in the availability and/or accessibility of food and/or presence of illnesses. We found that children in urban areas are more likely to be wasting than children in rural areas. The recent social changes and urbanization in PNG could explain this. The urban population has increased at an annual rate of 0.2% over the last decade, from 13.0% in 2010 to 13.25% in 2020 (28). The proportion of urban children in our study is higher (31%) because we included data from two urban sites in Madang and POM. Noticeably, the migration rates in our urban surveillance sites are also higher than the country average and many children in these sites reside in new resettlements (29). This could have been an important explanation for the high estimation of wasting among urban children. Earlier studies reported poor nutritional status among children in resettlement areas (8, 17).

Wasting among urban children is unlikely to be due to the lack of access to good quality foods, but more likely associated with poor breastfeeding practice and inappropriate food supplementation to CU5 (16). Most urban children aged 6–23 months are breastfed and feeding with supplementary foods is less than one half. This rate is even lower in POM and Madang. There are a number of reasons for suboptimal feeding practices among PNG women. Some are possibly culturally linked, for example, PNG men are advised not to have sex with their partners who are breastfeeding (12, 16). These men could have stopped their partners from breastfeeding their infants, contributing to the high level of wasting among CU5 in urban areas (8). Health education is therefore needed with interventions integrated into antenatal care and post-natal care and focused in urban areas to improve the knowledge of women and men about breastfeeding and food supplementation for their children, especially in the first 1,000 days of their life.

Household wealth is a good predictor of wasting. Cash crops are the main source of household income to contribute to and sustain household wealth in rural areas. Previous studies have found that wasting increases before the harvest and improves after major cash crops such as coffee, cocoa, coconut, and oil palm (30). We suggest that rural household wealth with the effects of cash crops are linked with consumption of good quality foods and both are important contributors to lowering the current wasting prevalence among CU5 in rural areas.

Cash crops are heavily dependent on seasonal effects. In PNG, climatic events such as drought and social conflicts such as tribal fights, possibly lead to acute food shortage at the household level (21). In this event, food supply is interrupted and reduces the quantity and quality of food, posing a threat to food security at the household level. In response to malnutrition, children will stop growing and lose body weight, including lean and fat tissues (31). This is another important seasonal aspect of PNG child wasting revealed in our data. Food insecurity in households is a key seasonal factor attributing to an increase in wasting among CU5 in PNG.

Household food shortage plays a large role in wasting among urban children in PNG. Around one-fifth of households experienced food shortage in the past 12 months. This is likely connected with social instabilities occurring in POM during the national election in 2017. Shops were closed, road traffic and transportation was blocked, and food supply chain in POM was broken. Job cuts led to a decline in households' affordability for foods. Serious droughts in the second half of 2017 resulted in the lost of subsistence farming throughout the country, but the most hard hit were those in the southern region. About 40% of households in Central Province reported food shortage in our data. Food security at the household level is an important seasonal factor, amplifying loss of weight-for-height, and contributing to the high wasting prevalence among CU5 in PNG. To effectively prevent wasting, food security must be addressed at the household level.

Previous studies have found that stunting is closely linked to poverty (15). This was also confirmed in our data. Stunting is often unrecognized because short stature is so common in some communities, particularly in the rural areas of PNG (32, 33). Our data show that children in Central Province, EHP, and ENB have a consistently higher risk of stunting than those in POM. Early nutritional surveys showed that a higher prevalence of stunting was related to the low protein and energy content of typical PNG diets (34, 35). In most of the rural areas of PNG, up to 80% of the total dietary energy comes from root crops such as yams, kaukau, and tapioca, which are high in fiber and moisture (12, 36).

Local factors maybe also contribute to stunting in children. Children in the highlands region, i.e., EHP, are more stunted as they are more involved in subsistence agriculture, which is connected with a higher energy expenditure and therefore with a need for more food. In EHP, infectious diseases such as respiratory tract infections, skin infections, and diarrhea are prevalent during the wet season and may have led to a higher level of stunting in this province (16, 25). By contrast, the frequent rainfall deficit along the coastal areas of the Southern region could have resulted in a persistent shortage of quality foods in Central Province. Lack of parental care during crop season and greater involvement of women in non-agriculture economic sectors such as trading and services might have also contributed to higher stunting among children in this province (21). Yams are one of the main foods in ENB. Studies on traditional food consumption in PNG showed strong associations between eating yams and stunting (12, 15, 30). Unlike other root vegetables, yams are harvested only once a year, often in the dry season, in the second half of the year and can be stored in households for a short period. This dietary factor could have contributed to high stunting among CU5 in ENB as shown in our data.

At the individual level, our data show that maternal socioeconomic status appears to be an important factor in the wasting and stunting of children. Women's marital status is a proxy for generational influence with polygamy widely accepted. Women's marital status could reflect their socioeconomic status in the household. In most rural areas of PNG, men are the ones who clear the bush and establish the gardens while women do gardening and food gathering (21). Single mothers might not be able to do the agriculture work needed to generate enough food to feed their children. Similarly, in urban areas, men make up the main labor force generating income for the household. Children whose fathers have multiple sexual partners might not have been adequately fed or attended to by their parents.

Maternal education appears to be a strong predictor for wasting and stunting among PNG CU5. Children whose mother had primary education were less likely to be wasted or stunted than children whose mothers had college/university level education. Previous study has found that children living in households where the household heads had a higher level of education were less likely to be malnourished (12). Our finding is not contradictory because most household heads are men rather than women regardless of their educational level. Higher educated mothers are more likely to participate in the official labor market and in full-time employment in the public or private sector, rather than doing housework. While the early learning system and childcare centers are not common in PNG, particularly in rural areas, more educated women often hire young women from their villages to take care of their children. However, these young babysitters often do not have adequate knowledge and experience to look after a child. Many infants are reportedly bottle fed (16), which may be explained by the higher education level of their mothers. Nutritional programs should include education intervention targeting child caretakers who work either in households or in childcare centers in order to reduce and prevent wasting and stunting among PNG children.

There are some limitations in this study. Child participants were recruited from CHESS surveillance sites located in the five main provinces, representing the four geographic regions of PNG. Hence, the data are not a nationally representative sample of the CU5 population of PNG. Information of maternal age, education level attainment and marital status, and household experience of food shortage in the past 12 months are self-reported by parents and caregivers, meaning the data might have been biased due to recall process. Furthermore, these covariates might have correlated with each other in the multinomial logistic regression models, resulted in collinearity and over-adjustment of ORs. Anthropometric data provide a profile of child participants' nutritional status, i.e., prevalence of wasting, stunting, being underweight, and overweight in child participants only at the data collection time. The data do not show the duration of such nutritional conditions. Lastly, a large proportion of child participants were excluded from statistical modeling because of missing value data on age and/or sex, and/or anthropometric measurement.

## Conclusion

This study has provided an update on nutritional status and household and maternal socioeconomic demographic factors associated with wasting and stunting among children under 5 years of age in PNG. There is no unique and clear explanation for the current status of wasting and stunting among PNG children. The best possible explanations in the context of contemporary PNG include the recent socioeconomic development leading to changes in the society, especially in urbanization associated with household wealth and in maternal education which have resulted in a shift in food habits and eating behaviors among children from a traditional low calorie diet to a diet which includes more high-calorie processed foods. This transition is triggered by various seasonal effects, from extreme climates to food shortage in the household and morbidity of child individuals. This transition is more apparent in urban areas, where children are likely exposed to more risk factors. These findings could be useful to inform policy and interventions in order to improve child nutrition among children in PNG. The study results provide evidence for further interventional studies of child health and development.

## Data Availability Statement

The raw data supporting the conclusions of this article will be made available by the authors, without undue reservation.

## Ethics Statement

The studies involving human participants were reviewed and approved by The CHESS was granted ethics approvals from Internal Review Board of PNG Institute of Medical Research (IRB's Approval No. 18.05) and the Medical Research Advisory Committee of Papua New Guinea (MRAC's Approval No. 18.06). These approvals covered all the data components under the CHESS, including data of children under 5 years of age, which were used in this manuscript. Informed consent was sought from self-identified household heads and woman participants. Women were informed about their right to withdraw from the study at any stage. Written informed consent to participate in this study was provided by the participants' legal guardian/next of kin.

## Author Contributions

BP designed the CHESS and analyzed the data, conceptualized the paper, interpreted the data, drafted, revised, finalized, and submitted the manuscript. VS supervised the fieldwork, collected data, and provided the inputs. AO reviewed, commented, and provided inputs, particularly on the English edition. WP oversaw the CHESS and approved the submission of the manuscript. All authors contributed to the article and approved the submitted version.

## Conflict of Interest

The authors declare that the research was conducted in the absence of any commercial or financial relationships that could be construed as a potential conflict of interest.
